# Treatment of Hysterical Mania

**Published:** 1872-05

**Authors:** J. Crichton Browne

**Affiliations:** Medical Director, West Riding Asylum; Lecturer on Mental Diseases to the Leeds School of Medicine, &c.


### TREATMENT OF HYSTERICAL MANIA.

By J. Crichton Browne, M. D., F. R. S. E., Medical Director, West Riding Asylum; Lecturer on Mental Diseases to the Leeds School of Medicine, &c.

The treatment of hysterial mania, Dr. Browne writes, requires a careful adaption to varying conditions. Its occasionally transitory nature justifies a trial of home treatment before an asylum is resorted to; but if under home treatment it remains unabated at the end of fourteen days, then removal should not be any longer delayed. The most protracted and troublesome cases that have fallen under Dr. Browne's observation have been those in which home treatment had been persevered in for months, until patience had been exhausted. The prolongation of the disease increases the risk of relapse, so that it is of much importance to cut it short at the earliest possible moment. During the maniacal condition there is not much room for moral treatment. A conciliatory and yet firm manner on the part of the physician, however, is not without its effect. Quietness and rest are also advantageous, and any simple occupation, such as sewing, if its adoption can be secured during an interval of tranquility, is often very useful. It fixes attention, and by its very monotony soothes the perturbed mind. Exercise in the fresh air ought to be taken daily, and nourishing food must be administered. The medical treatment Dr. Browne generally begins with is a mixture containing bromide of potassium and tincture of valerian—forty grains of the former and a drachm of the latter in each dose, to be taken three or four times a day. This has sometimes a most gratifying effect; if, however, its beneficial action be not speedily manifested, no good will result from continuing its employment. Dr. Browne recommends us, then, to resort to morphia and assafœtida. From a quarter to half a grain of muriate of morphia, with from ten to forty grains of assafœtida, may be given twice or thrice a day. The tincture of assafœtida is not objected to in pauper asylums. This treatment is generally successful; but should it fail, then cannabis indica with bromide of potassium ought to be tried. The use of narcotics is not contra-indicated in hysterical mania. Warm, tepid and even cold shower-baths are sometimes composing and useful. During convalescence, iron is most always required; sometimes quinine also. Dr. Browne has a particularly high opinion of the value of Easton's syrup of the phosphates of iron, quinine, and strychnia, during recovery from hysterical mania. Of course, menstrual disorders must be subjected to their appropriate treatment.—*British Medical Journal—Half-Yearly Abstract.*

